# Advanced lung cancer inflammation index combined with geriatric nutritional risk index predict all-cause mortality in heart failure patients

**DOI:** 10.1186/s12872-023-03608-x

**Published:** 2023-11-17

**Authors:** Tao Shi, Yan Wang, Yunzhu Peng, Meifen Wang, Yanji Zhou, Wenyi Gu, Yanyan Li, Jie Zou, Na Zhu, Lixing Chen

**Affiliations:** 1 The First Affiliated Hospital of Kunming Medical University, Kunming, China; 2 Kunming Children’s Hospital, Kunming, China

**Keywords:** Heart failure, Advanced lung cancer inflammation index, Geriatric nutritional risk index, Nutrition, Inflammation

## Abstract

**Background:**

This study was undertaken to explore the predictive value of the advanced lung cancer inflammation index (ALI) combined with the geriatric nutritional risk index (GNRI) for all-cause mortality in patients with heart failure (HF).

**Methods and results:**

We enrolled 1123 patients with HF admitted to our cardiology department from January 2017 to October 2021. Patients were divided into four groups, according to the median ALI and GNRI. From the analysis of the relationship between the ALI and GNRI, we concluded that there was a mild positive linear correlation (*r* = 0.348, *p* < 0.001) and no interaction (*p* = 0.140) between the ALI and GNRI. Kaplan‒Meier analysis showed that the cumulative incidence of all-cause mortality in patients with HF was highest in Group 1 (log-rank χ^2^ 126.244, *p* < 0.001). Multivariate Cox proportional hazards analysis revealed that ALI and GNRI were independent predictors of all-cause mortality in HF patients (ALI: HR 0.407, 95% CI 0.296–0.560, *p* < 0.001; GNRI: HR 0.967, 95% CI 0.954–0.980, *p *< 0.001). The area under the curve (AUC) for ALI combined with GNRI was 0.711 (*p* < 0.001), according to the time-dependent ROC curve.

**Conclusion:**

ALI and GNRI were independent predictors of all-cause mortality in HF patients. Patients with HF had the highest risk of all-cause mortality when the ALI was < 24.60 and the GNRI was < 94.41. ALI combined with the GNRI has good predictive value for the prognosis of HF patients.

## Introduction

Heart failure (HF) is the major cause of morbidity and mortality worldwide and is on the increase [1]. China is the most populous country in the world, so the total number of HF patients is enormous. The incidence and cost of treatment for HF patients are likely to increase in the next decade due to the ageing population [2]. HF imposes a huge financial burden on patients and is a significant resource drain on health care systems, and HF is becoming a global problem [3].

Patients with HF are characterized by high levels of inflammation, hypercatabolic syndrome with weight loss, skeletal sarcopenia and muscle wasting, and clinically, these patients are often severely malnourished because nutritional interventions are often lacking or neglected [4]. Malnutrition in HF patients sometimes progresses to overt "cardiac cachexia", characterized by protein-calorie malnutrition with muscle atrophy and peripheral oedema [5]. Although such extreme states may be apparent, recognizing less severe malnutrition can be more difficult. It is therefore necessary to carry out a routine assessment of the nutritional status of patients with HF.

Several objective nutritional indices are often used in the HF field, among which the geriatric nutritional risk index (GNRI), controlling nutritional status (CONUT) score and prognostic nutrition index (PNI) are recognized prognostic markers in patients with HF [6–8]. Among these, the GNRI appears to be a relatively optimal tool for screening malnutrition and showed the highest prognostic value in outpatients with HF [6]. The advanced lung cancer inflammation index (ALI) is an emerging inflammatory and nutritional biomarker that includes neutrophil-to-lymphocyte ratio (NLR), body mass index (BMI), and serum albumin. Several studies have reported the predictive value of ALI on the prognosis of patients with HF, with smaller values of ALI implying a worse prognosis for the patient [9, 10]. When the ALI value is smaller (i.e., smaller BMI and Alb, larger NLR) it means that the patient is more malnourished and has a more severe inflammatory response. However, no studies have confirmed the predictive value of the ALI combined with the GNRI for the prognosis of patients with HF. Therefore, the aim of this study was to investigate the prognostic impact of the ALI combined with the GNRI in HF patients.

## Methods

### Study population

This is a retrospective study. We enrolled 1221 patients with HF who were admitted to the First Affiliated Hospital of Kunming Medical University for acute exacerbation of chronic HF between January 2017 and October 2021. We included patients admitted with HF classified as New York Heart Association (NYHA) functional class III or IV and a brain natriuretic peptide (BNP) level of ≥ 500 pg/mL. We included 1123 patients with HF in this study after excluding those who lacked the necessary data (e.g., routine blood tests, cardiac ultrasound data or BMI), had a combination of other serious diseases (e.g., malignancies, infectious diseases, blood diseases, or severe renal or liver dysfunction) or were lost to follow-up.

### Data collection

At the time of admission, demographic and clinical information, electrocardiograms, cardiac ultrasound data and blood samples were collected. Data included patient age and sex, heart rate (HR), blood pressure (BP), BMI, NYHA cardiac function classification, medical history, treatment history, BNP, white blood cells (WBC), red blood cells (RBC), neutrophils (NBC), lymphocytes (LBC), C-reactive protein (CRP), haemoglobin (Hb), platelets (PLT), sodium, potassium, chlorine, albumin (Alb), alanine aminotransferase (ALT), aspartate aminotransferase (AST), creatinine (Cre), uric acid (UA), total cholesterol (TC) and estimated glomerular filtration rate (eGFR), QRS width, left atrial diameter (LAd), left ventricular end-diastolic diameter (LVDd), right atrial diameter (RAd), right ventricular diameter (RVd), and left ventricular ejection fraction (LVEF). Blood samples were taken from all patients after an overnight fast (8–12 h) and sent to the laboratory of the First Affiliated Hospital of Kunming Medical University.

The following formula was used to derive the ALI: ALI = BMI × Alb/NLR, where BMI = weight in kilograms/(height in metres)^2^, Alb = serum albumin in grams per decilitre, and NLR = absolute neutrophil count/absolute lymphocyte count [11]. The GNRI was calculated according to the following formula: GNRI = 14.89 × Alb (g/dL) + 41.7 × BMI/22, where BMI/22 was set to 1 if the patient's BMI/22 was greater than 1 [12].

The researchers collected the survival data by conducting telephone interviews with the patients or their family members. Follow-up would end at the time of the patient's last available medical record if no response was received. All-cause mortality was the primary endpoint of the study.

### Statistical analysis

Patients with HF were divided into four groups according to the median ALI and GNRI: Group 1 (ALI < 24.60 and GNRI < 94.41), Group 2 (ALI < 24.60 and GNRI ≥ 94.41), Group 3 (ALI ≥ 24.60 and GNRI < 94.41) and Group 4 (ALI ≥ 24.60 and GNRI ≥ 94.41). When describing baseline patient characteristics, continuous variables are expressed as the mean ± standard deviation when normally distributed, otherwise as the median with interquartile range, while categorical variables are expressed as numbers and percentages. ALI and BNP were logarithmically transformed due to the highly skewed distribution of the original values. When comparing baseline characteristics between the four groups, variance analyses were used for normally distributed continuous variables, Mann‒Whitney U tests for nonnormally distributed data, and chi-square tests for categorical variables. Correlation analysis based on Spearman’s nonparametric test and two-way ANOVA interaction analysis were used to assess the correlation and interaction between the ALI and GNRI, respectively. To assess the relationship between the ALI and GNRI and all-cause mortality, we used Kaplan‒Meier curves for analysis and log-rank tests for comparison. Univariate Cox proportional hazard regression analyses were used to roughly show the effect of each variable on all-cause mortality. Subsequently, multivariate Cox proportional hazard regression analysis was performed on variables with *p* values less than 0.05 in the univariate analysis to identify independent predictors of all-cause mortality in patients with HF. Receiver operating characteristic (ROC) analysis was used to assess the predictive value of the ALI combined with the GNRI on the risk of all-cause mortality in patients with HF. This study used IBM SPSS Statistics version 26.0 and MedCalc for data analysis. A *p* value less than 0.05 was regarded as statistically significant.

## Results

### Baseline patient characteristics

In this study, we included 1123 patients with HF. Based on the median ALI and GNRI, we divided the patients into four groups: Group 1 (*n* = 336), Group 2 (*n* = 225), Group 3 (*n* = 221) and Group 4 (*n* = 341). We found that there were statistically significant differences between Group 1 and Group 4 in age, diastolic BP, BMI, NYHA class, coronary artery disease, lg BNP, WBC, RBC, NBC, LBC, NLR, CRP, Hb, sodium, potassium, chlorine, Alb, Cre, UA, TC, eGFR, QRS wave, LAd, LVDd, and LVEF (*p* < 0.05) (Table 1).

**Table 1:** Baseline characteristics according to ALI combined with GNRI

Variables	Total (*n* = 1123)	Group 1 (*n* = 336)	Group 2 (*n* = 225)	Group 3 (*n* = 221)	Group 4 (*n* = 341)	*p*
***Basic characteristics***						
Male	693 (61.7%)	219 (65.2%)	140 (62.2%)	125 (56.6%)	209 (61.3%)	0.237
Age(year)	66.82 ± 12.58	70.31 ± 11.71	68.34 ± 11.16	64.57 ± 13.36	63.83 ± 12.78	< 0.001
HR(beat/minute)	85.46 ± 21.17	87.62 ± 22.59	86.12 ± 20.68	83.39 ± 19.53	84.23 ± 20.94	0.073
Systolic BP(mmHg)	121.87 ± 22.83	121.44 ± 23.64	120.68 ± 21.61	122.14 ± 20.80	122.93 ± 24.07	0.683
Diastolic BP(mmHg)	76.24 ± 15.04	74.06 ± 14.68	76.18 ± 14.72	76.53 ± 14.80	78.23 ± 15.51	0.004
BMI(kg/m2)	22.98 ± 3.85	21.45 ± 3.36	23.55 ± 3.04	22.65 ± 4.49	24.31 ± 3.77	< 0.001
NYHA class IV	418 (37.2%)	144 (42.9%)	95 (42.2%)	68 (30.8%)	111 (32.6%)	0.003
***Medical history***						
Coronary artery disease	574 (51.1%)	174 (51.8%)	135 (60.0%)	96 (43.4%)	169 (49.6%)	0.005
Hypertension	620 (55.2%)	193 (57.4%)	127 (56.4%)	114 (51.6%)	186 (54.5%)	0.562
Diabetes	315 (28.0%)	113 (33.6%)	56 (24.9%)	56 (25.3%)	90 (26.4%)	0.056
Atrial fibrillation	383 (34.1%)	110 (32.7%)	72 (32.0%)	80 (36.2%)	121 (35.5%)	0.696
Stroke	158 (14.1%)	60 (17.9%)	29 (12.9%)	30 (13.6%)	39 (11.4%)	0.100
***Treatment***						
SGLT-2I	252 (22.4%)	74 (22.0%)	52 (23.1%)	58 (26.2%)	68 (19.9%)	0.369
β-receptor blockers	786 (70.0%)	226 (67.3%)	153 (68.0%)	159 (71.9%)	248 (72.7%)	0.357
Diuretics	883 (78.6%)	261 (77.7%)	173 (76.9%)	176 (79.6%)	273 (80.1%)	0.769
ACEI/ARB/ARNI	627 (55.8%)	193 (57.4%)	128 (56.9%)	118 (53.4%)	188 (55.1%)	0.788
CRT/CRTD	111 (9.9%)	31 (9.2%)	28 (12.4%)	19 (8.6%)	33 (9.7%)	0.523
***Laboratory indicators***						
LgBNP	3.17 ± 0.28	3.23 ± 0.29	3.16 ± 0.28	3.14 ± 0.25	3.14 ± 0.28	< 0.001
WBC(10^9/L)	6.87 (5.53, 9.01)	7.69 (6.09, 10.85)	8.47 (6.56, 10.92)	6.26 (5.16, 7.53)	6.21 (5.21, 7.49)	< 0.001
RBC(10^12/L)	4.55 ± 0.77	4.32 ± 0.79	4.59 ± 0.68	4.59 ± 0.79	4.71 ± 0.74	< 0.001
NBC(10^9/L)	4.54 (3.51, 6.52)	5.61 (4.24, 8.46)	6.62 (4.81, 8.46)	3.70 (2.86, 4.50)	3.85 (3.16, 4.65)	< 0.001
LBC(10^9/L)	1.38 (1.03, 1.81)	1.07 (0.77, 1.37)	1.08 (0.81, 1.41)	1.82 (1.43, 2.18)	1.63 (1.34, 2.06)	< 0.001
NLR	3.31 (2.24, 5.47)	5.21 (3.83, 8.12)	5.81 (4.52,8.27)	2.07 (1.61, 2.62)	2.37 (1.87, 2.83)	< 0.001
CRP (mg/L)	7.50 (2.90, 22.00)	15.50 (5.73, 39.26)	10.30 (3.51, 25.50)	5.85 (2.57, 15.55)	4.88 (2.08, 12.20)	< 0.001
Hb(g/L)	138.35 ± 23.93	131.44 ± 25.88	139.42 ± 21.37	139.73 ± 24.88	143.54 ± 21.28	< 0.001
PLT(10^9/L)	191.00 (148.00, 242.00)	191.50 (137.00, 245.00)	194.00 (142.50, 243.50)	192.00 (147.00, 249.00)	187.00 (155.00, 235.50)	0.988
Sodium(mmol/L)	141.03 ± 4.48	140.41 ± 5.08	140.09 ± 4.48	141.49 ± 4.39	141.98 ± 3.62	< 0.001
Potassium(mmol/L)	3.94 ± 0.59	3.89 ± 0.64	3.98 ± 0.64	3.89 ± 0.60	3.99 ± 0.50	0.041
Chlorine(mmol/L)	102.90 ± 4.69	102.38 ± 4.95	101.75 ± 5.06	103.70 ± 4.55	103.64 ± 4.00	< 0.001
Alb(g/dL)	3.67 ± 0.45	3.28 ± 0.37	3.94 ± 0.30	3.44 ± 0.26	4.01 ± 0.30	< 0.001
ALT(IU/L)	25.00 (16.70, 42.30)	24.75 (15.93, 43.13)	25.60 (17.05, 44.75)	25.30 (16.70, 45.65)	25.00 (17.00, 41.00)	0.859
AST(IU/L)	28.90 (20.20, 44.00)	30.30 (20.00, 50.40)	29.00 (20.80, 49.00)	29.00 (20.15, 39.90)	27.10 (20.15, 39.85)	0.106
Cre(umol/L)	103.30 (83.20, 134.10)	110.85 (84.85, 154.80)	108.60 (88.95, 139.00)	95.40 (79.05, 117.70)	101.30 (82.95, 123.65)	< 0.001
UA(umol/L)	480.20 (374.10, 591.40)	471.95 (346.78, 582.20)	491.60 (383.60, 613.40)	481.00 (364.80, 574.65)	491.85 (395.23, 596.33)	0.047
TC(mmol/L)	3.67 ± 1.00	3.45 ± 1.00	3.81 ± 1.07	3.65 ± 0.97	3.79 ± 0.95	< 0.001
eGFR(ml/min)	43.79 (32.21, 56.34)	36.91 (25.33, 50.17)	40.92 (31.52, 52.24)	47.24 (36.29, 61.58)	49.75 (36.49, 61.63)	< 0.001
***ECG parameters and cardiac ultrasound index***						
QRS wave(ms)	106.00 (94.00, 128.00)	104.00 (90.00, 126.00)	112.00 (96.00, 136.00)	104.00 (94.00, 128.00)	107.00 (95.00, 127.00)	0.011
LAd(mm)	42.42 ± 9.45	40.21 ± 9.44	43.14 ± 9.48	42.63 ± 9.35	43.93 ± 9.13	< 0.001
LVDd(mm)	56.20 ± 12.67	53.09 ± 12.20	56.87 ± 12.07	56.00 ± 11.14	58.88 ± 13.77	< 0.001
RAd(mm)	52.14 ± 12.62	51.34 ± 13.16	51.28 ± 12.90	52.87 ± 12.73	52.99 ± 11.77	0.202
RVd(mm)	67.63 ± 16.30	66.56 ± 16.49	67.16 ± 15.65	67.74 ± 15.92	68.89 ± 16.75	0.308
LVEF(%)	43.00 (33.00, 58.00)	46.50 (35.00, 60.00)	43.00 (33.00, 56.00)	45.00 (33.00, 59.00)	40.00 (31.00, 54.00)	< 0.001

① Differences in normally distributed continuous variables were compared using variance analyses, and those in nonnormally distributed data were compared using Mann‒Whitney U tests. Chi-square tests were used to compare differences in categorical variables between groups. *P* values are derived from comparing the Group 1 and Group 4. *P* < 0.05 was considered indicative of statistical significance

② *HR* heart rate, *BP* blood pressure, *BMI* body mass index, *NYHA* New York Heart Association, *SGLT-2I* sodium-glucose cotransporter 2 inhibitor, *ACEI* angiotensin converting enzyme inhibitor, *ARB* angiotensin II receptor blocker, *ARNI* angiotensin receptor-enkephalinase inhibitor, *CRT* cardiac resynchronisation therapy, *CRTD* Cardiac Resynchronisation Therapy Defibrillator, *BNP* brain natriuretic peptide, *WBC* white blood cells, *RBC* red blood cells, *NBC* neutrophil, *LBC* lymphocyte, *NLR* neutrophil-to-lymphocyte ratio, *CRP* C-reactive protein, *Hb* haemoglobin, *PLT* platelet, *Alb* albumin, *ALT* alanine aminotransferase, *AST* aspartate aminotransferase, *Cre* creatinine, *UA* uric acid, *TC* total cholesterol, *eGFR* estimated glomerular filtration rate, *LAd* left atrium diameter, *LVDd* left ventricular end-diastolic diameter, *RAd* right atrium diameter, *RVd* right ventricle diameter, *LVEF* left ventricular ejection fraction

### ALI and GNRI

**Fig. 1:**
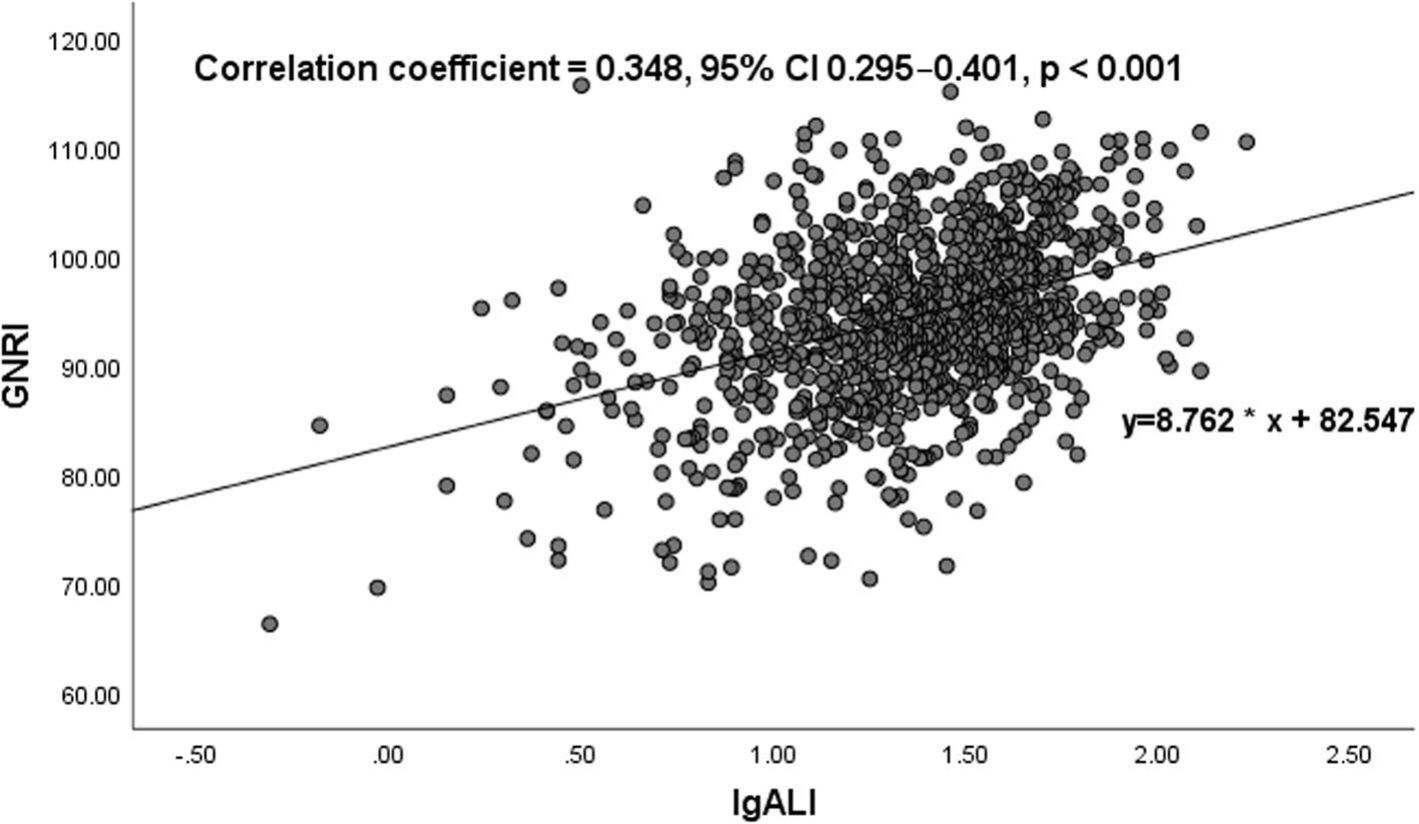
Correlation between advanced lung cancer inflammation index (ALI) and geriatric nutritional risk index (GNRI). The ALI was mildly positively linearly correlated with the GNRI (correlation coefficient = 0.348, 95% confidential interval (CI) 0.295–0.401, *p* < 0.001)

**Fig. 2:**
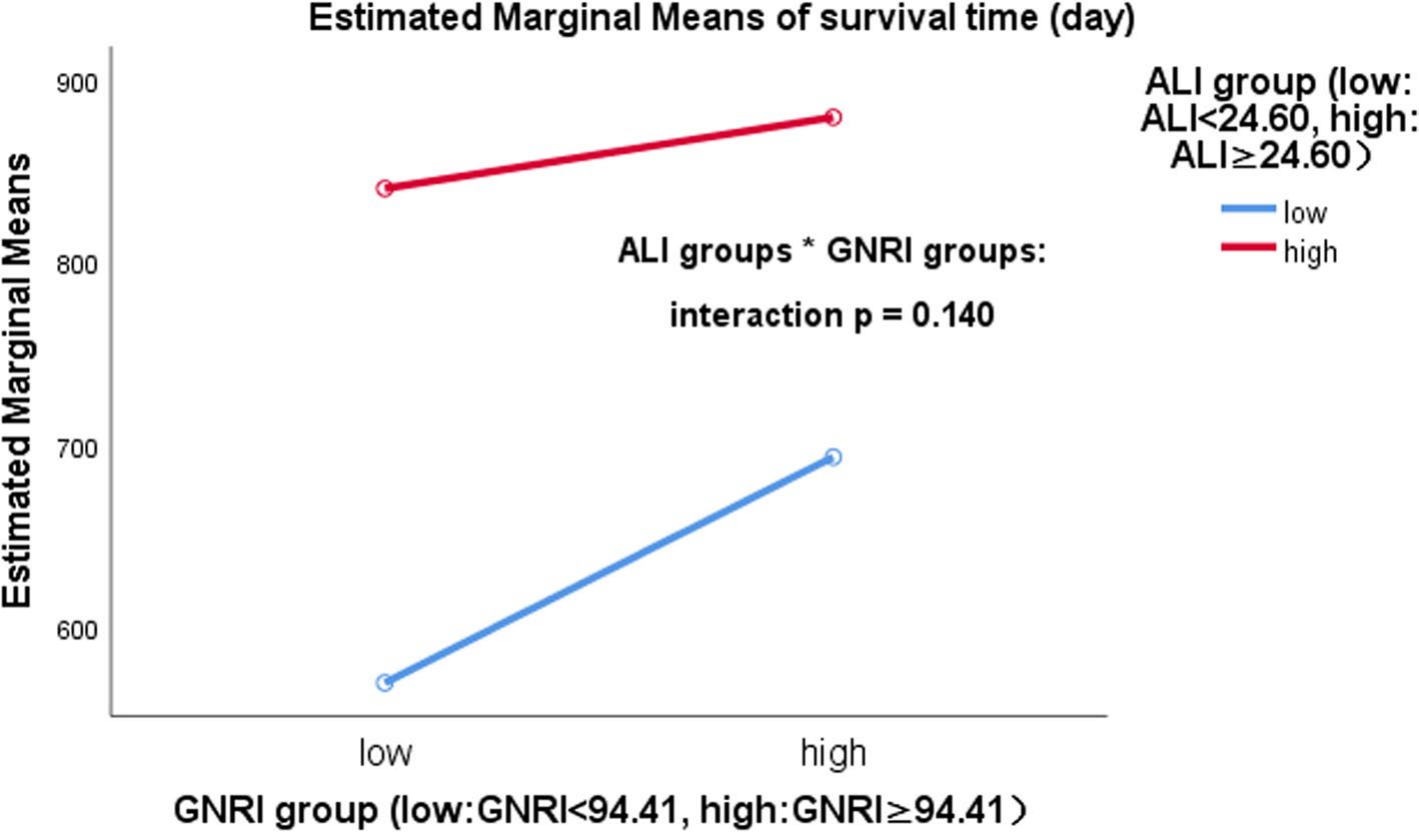
Interaction between advanced lung cancer inflammation index (ALI) and geriatric nutritional risk index (GNRI). There was no interaction between the ALI and GNRI (interaction *p* = 0.140)

The GNRI is widely used in nutritional assessment, and the ALI is a novel inflammatory and nutritional biomarker. Therefore, we examined the correlation of the ALI and GNRI in HF patients. A correlation analysis based on Spearman’s nonparametric test showed that the ALI was mildly positively linearly correlated with the GNRI in the total study population. [Spearman’s correlation coefficient (r): 0.348, *p* < 0.001] (Fig. 1). Two-way ANOVA revealed no interaction between the ALI and GNRI (*p* = 0.140) (Fig. 2).

### Association between ALI and GNRI and all-cause mortality

**Fig. 3:**
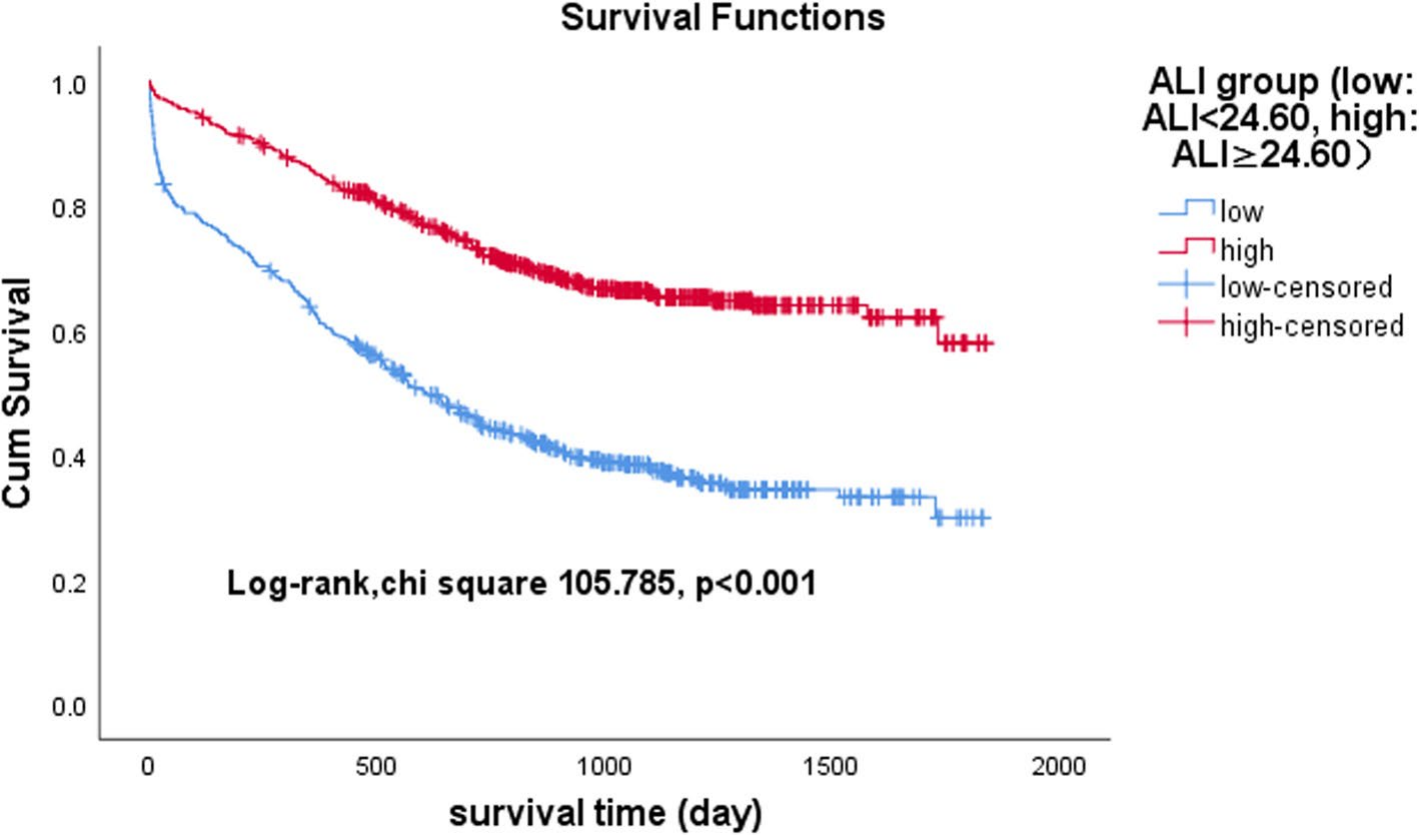
Kaplan‒Meier survival curves for heart failure patients across the advanced lung cancer inflammation index (ALI)

**Fig. 4:**
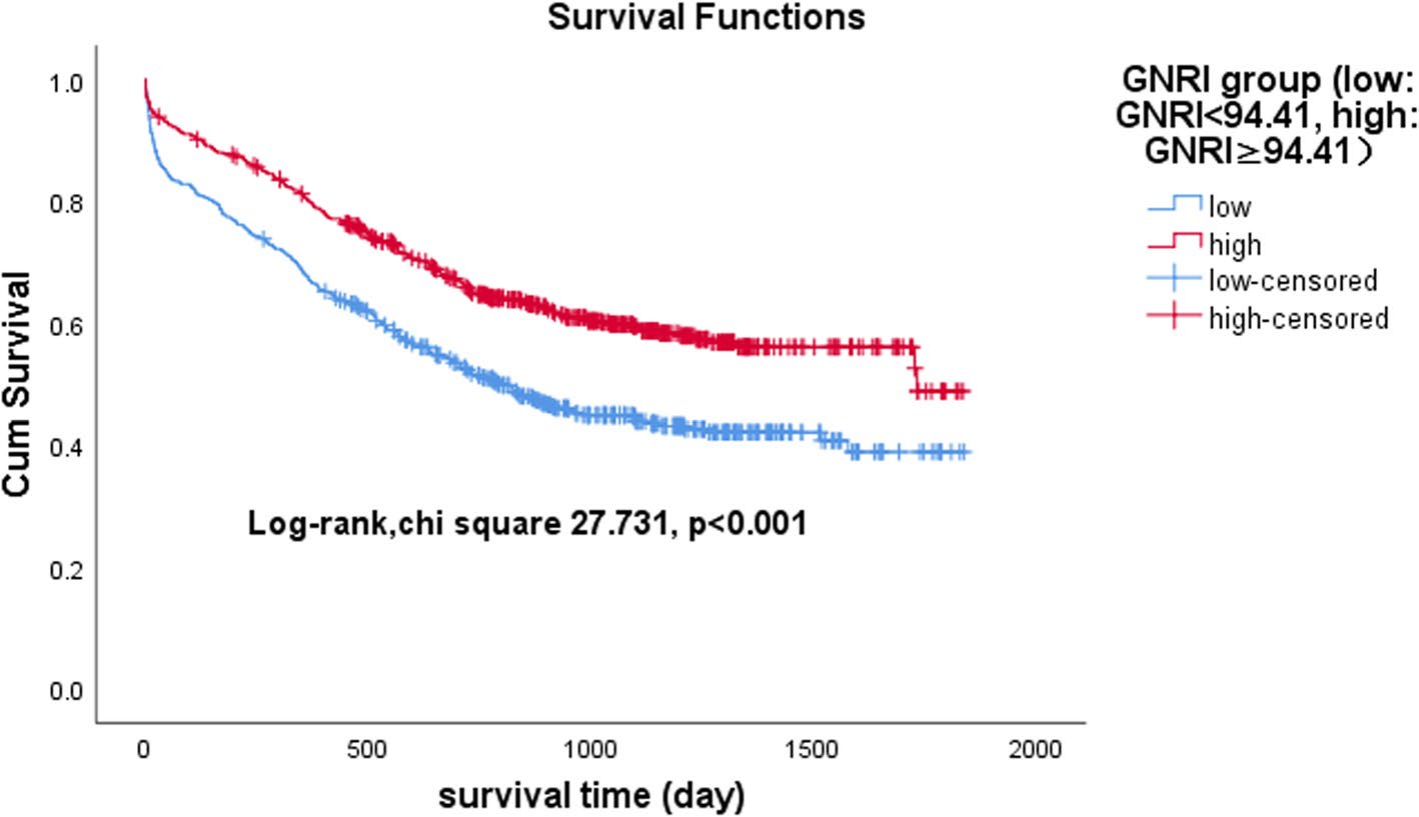
Kaplan‒Meier survival curves for heart failure patients across the geriatric nutritional risk index (GNRI)

**Fig. 5:**
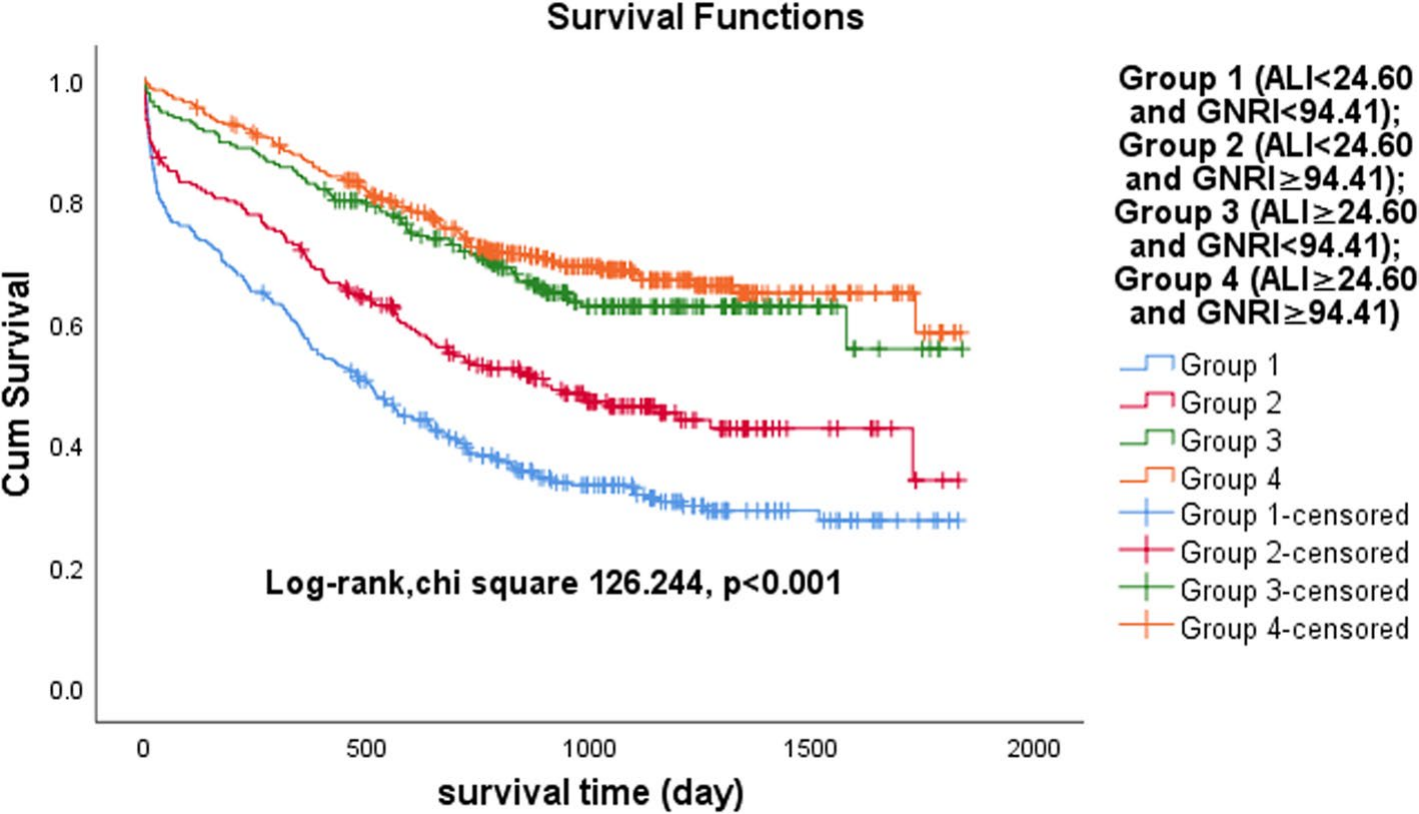
Kaplan‒Meier survival curves for heart failure patients in the four groups. Group 1 (ALI < 24.60 and GNRI < 94.41); Group 2 (ALI < 24.60 and GNRI ≥ 94.41); Group 3 (ALI ≥ 24.60 and GNRI < 94.41); Group 4 (ALI ≥ 24.60 and GNRI ≥ 94.41). ALI, advanced lung cancer inflammation index; GNRI, geriatric nutritional risk index

To test the prognostic significance of the ALI combined with GNRI in patients with HF, we conducted a Kaplan‒Meier analysis. The Kaplan‒Meier analysis showed that the cumulative incidence of all-cause death was significantly higher in HF patients in the low group than in those in the high group, either in the ALI group or the GNRI group (ALI: log-rank χ^2^ 105.785, *p* < 0.001; GNRI: log-rank χ^2^ 27.731, *p* < 0.001) (Figs. 3 and 4). When the ALI was combined with GNRI, the Kaplan‒Meier analysis revealed that the cumulative incidence of all-cause mortality in HF patients was highest in Group 1, second highest in Group 2 and tied for lowest in Group 3 and Group 4 (log-rank χ^2^ 126.244, *p* < 0.001) (Fig. 5).

### ALI and GNRI as predictors of adverse outcomes

After correcting for age, NYHA class, heart rate, systolic BP, diastolic BP, WBC, CRP, ALT, AST, uric acid, eGFR, sodium, chloride, and lg BNP, the multivariate Cox proportional hazards analyses showed that the ALI and GNRI were independent predictors of all-cause death in patients with HF. (ALI: HR 0.407, 95% CI 0.296–0.560, *p* < 0.001; GNRI: HR 0.967, 95% CI 0.954–0.980, *p* < 0.001) (Table 2). Hazard ratios for mortality in patients with HF based on the median ALI and GNRI were calculated. The results show that Group 1 always has the highest risk of death, whether it is Model 1, Model 2 or Model 3, using Group 4 as a reference (Table 3). We can conclude that the risk of all-cause mortality in patients with HF is highest when the ALI is < 24.60 and GNRI < 94.41.

**Table 2:** Results of univariable analysis and final model of multivariable analysis using Cox proportional hazard analysis of all-cause death

	**Univariable**		**Multivariable**	
	HR (95% CI)	*p*	HR (95% CI)	*p*
Age	1.033 (1.026, 1.041)	< 0.001	1.021 (1.013, 1.030)	< 0.001
Female (reference: male)	0.972 (0.815, 1.160)	0.756		
NYHA class (reference: class IV)	0.410 (0.345, 0.487)	< 0.001	0.551 (0.458, 0.663)	< 0.001
Heart rate	1.008 (1.004, 1.012)	< 0.001		
LVEF	0.995 (0.990, 1.001)	0.089		
Systolic BP	0.994 (0.990, 0.998)	0.002		
Diastolic BP	0.985 (0.979, 0.991)	< 0.001	0.992 (0.985, 0.998)	0.011
WBC	1.072 (1.051, 1.093)	< 0.001		
CRP	1.012 (1.010, 1.014)	< 0.001	1.008 (1.005, 1.010)	< 0.001
ALT	1.003 (1.002, 1.004)	< 0.001		
AST	1.005 (1.004, 1.005)	< 0.001	1.002 (1.001, 1.004)	< 0.001
Uric acid	1.002 (1.001, 1.002)	< 0.001	1.001 (1.000, 1.001)	0.002
eGFR	0.973 (0.968, 0.978)	< 0.001		
Sodium	0.937 (0.918, 0.955)	< 0.001		
Potassium	1.143 (0.986, 1.325)	0.077		
Chlorine	0.930 (0.913, 0.947)	< 0.001	0.972 (0.954, 0.991)	0.003
Lg BNP	5.754 (4.185, 7.911)	< 0.001	3.008 (2.130, 4.250)	< 0.001
GNRI	0.948 (0.937, 0.959)	< 0.001	0.967 (0.954, 0.980)	< 0.001
Lg ALI	0.139 (0.109, 0.178)	< 0.001	0.407 (0.296, 0.560)	< 0.001

① Corrected for age, NYHA class, heart rate, systolic BP, diastolic BP, ALT, AST, creatinine, uric acid, eGFR, sodium, chloride, lg BNP

② *HR* hazard ratio, *CI* confidence interval, *NYHA* New York Heart Association, *LVEF* left ventricular ejection fraction, *BP* blood pressure, *WBC* white blood cells, *CRP* C-reactive protein, *ALT* alanine aminotransferase,* AST* aspartate aminotransferase, *eGFR* estimated glomerular filtration rate, *BNP* brain natriuretic peptide, *GNRI* geriatric nutritional risk index, *ALI* advanced lung cancer inflammation index

**Table 3:** Hazard ratios for mortality of patients with heart failure according to the medians of ALI and GNRI

	**Unadjusted**		**Model 1**		**Model 2**		**Model 3**	
**Groups**	**HR (95% CI)**	***p***	**HR (95% CI)**	***p***	**HR (95% CI)**	***p***	**HR (95% CI)**	***p***
Group 4 (*n* = 264)	1		1		1		1	
Group 1 (*n* = 264)	3.113 (2.468, 3.926)	< 0.001	2.656 (2.096–3.366)	< 0.001	2.616 (2.064–3.316)	< 0.001	2.056 (1.604–2.635)	< 0.001
Group 2 (*n* = 177)	2.067 (1.589, 2.689)	< 0.001	1.880 (1.444–2.449)	< 0.001	1.817 (1.395–2.368)	< 0.001	1.264 (0.954–1.673)	0.102
Group 3 (*n* = 177)	1.166 (0.869,1.564)	0.307	1.130 (0.842,1.517)	0.416	1.169 (0.871,1.570)	0.298	1.244 (0.923,1.677)	0.151

① Group 1, Group 2 and Group 3 all used Group 4 as the reference group

② Group 1 (ALI < 24.60 and GNRI < 94.41); Group 2 (ALI < 24.60 and GNRI ≥ 94.41); Group 3 (ALI ≥ 24.60 and GNRI < 94.41); Group 4 (ALI ≥ 24.60 and GNRI ≥ 94.41)

③ Model 1: adjusted for age; Model 2: adjusted for age, NYHA class and diastolic BP; Model 3: adjusted for Model 2 + CRP, AST, uric acid, chloride, lg BNP

④ *ALI* advanced lung cancer inflammation index, *GNRI* geriatric nutritional risk index, *HR* hazard ratio, *CI* confidence interval, *NYHA* New York Heart Association, *BP* blood pressure, *CRP* C-reactive protein, *AST* aspartate aminotransferase, *BNP* brain natriuretic peptide

### Predictive ability of the ALI combined with the GNRI in patients with HF

**Fig. 6:**
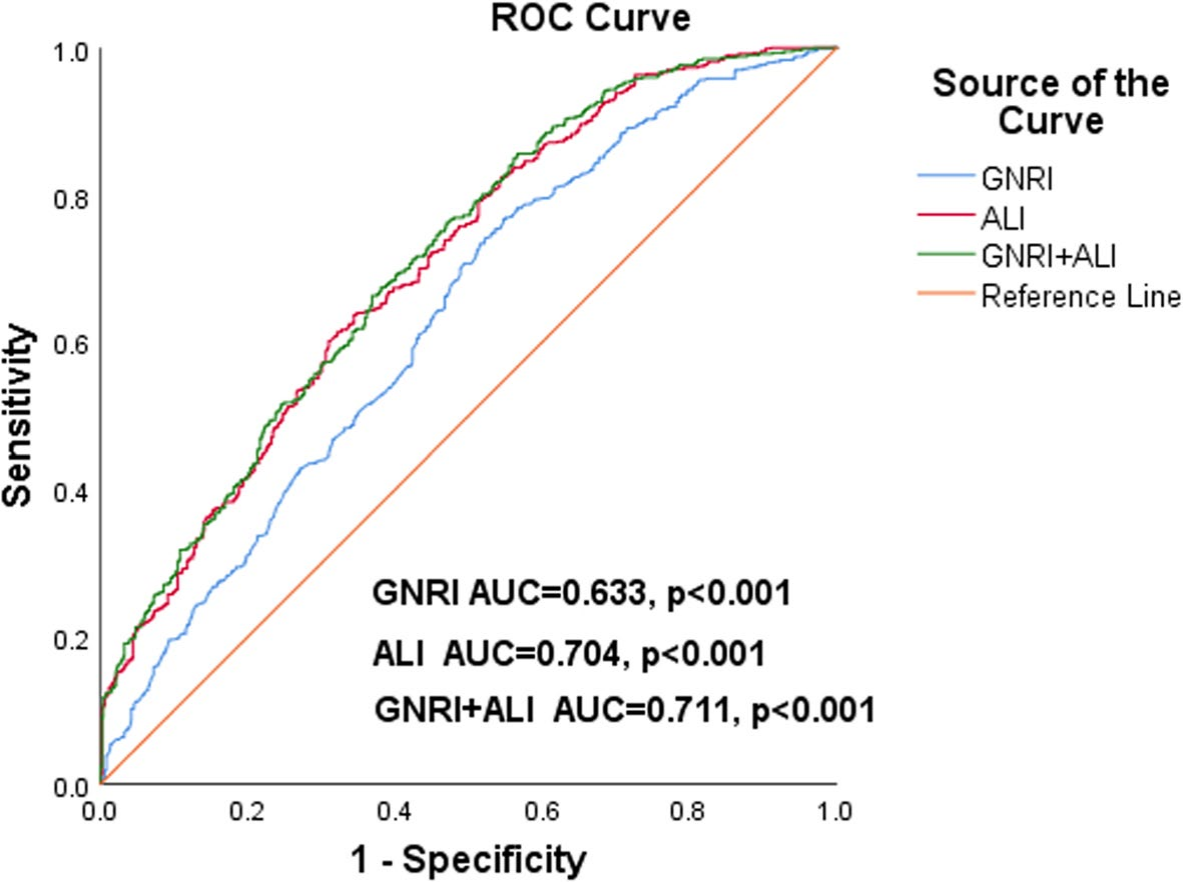
Time-dependent receiver operating characteristic (ROC) curves of advanced lung cancer inflammation index (ALI), geriatric nutritional risk index (GNRI) and GNRI + ALI with reference line for all-cause mortality in heart failure patients. AUC, area under the curve

The time-dependent ROC curves showed that the area under the curve (AUC) was 0.633 for GNRI (*p* < 0.001), 0.704 for ALI (*p *< 0.001) and 0.711 for ALI combined with GNRI (*p* < 0.001) (Fig. 6). Based on the above analyses, we can conclude that the ALI combined with the GNRI had good predictive power for the prognosis of HF patients.

## Discussion

This retrospective observational study was conducted to demonstrate that the ALI combined with the GNRI has a significant impact on the prognosis of patients with HF. Multivariate Cox proportional hazards analysis revealed that both ALI and GNRI were independent predictors of all-cause mortality in HF patients (ALI: HR 0.407, 95% CI 0.296–0.560, *p* < 0.001; GNRI: HR 0.967, 95% CI 0.954–0.980, *p* < 0.001). The hazard ratios for mortality in the four groups showed that Group 1 always has the highest risk of all-cause mortality, using Group 4 as a reference. The time-dependent ROC curves revealed that the AUC for the ALI combined with the GNRI was 0.711 (*p* < 0.001), with a sensitivity of 67.34% and a specificity of 61.98%. The ALI combined with the GNRI has good predictive value for prognosis in HF patients.

Factors contributing to HF fall into four categories: first, common risk factors such as hypertension, ischaemic injury and metabolic syndrome; second, inherited heart disease such as hypertrophic cardiomyopathy; third, mechanical changes such as valve dysfunction; and finally, immune-related causes including bacterial and viral infections and autoimmune reactions [13]. The calculation of ALI involves NLR, BMI and Alb, and GNRI is calculated from BMI and Alb. Of these, BMI and Alb are both common clinical markers that can reflect a patient's nutritional status to some extent. There is a strong link between HF and malnutrition. Malnutrition can lead to hypoproteinaemia and a weakened immune system, which can aggravate HF and infections [14]. In contrast, as HF worsens, patients' nutritional status deteriorates due to bowel oedema and reduced food intake, resulting in a vicious cycle [15–17]. However, obesity may also cause HF mainly because of the haemodynamic changes associated with activation of the renin–angiotensin–aldosterone system, enhanced sympathetic nervous system activity and mineralocorticoid receptor expression, and production of acute phase proteins and inflammatory cytokines [18].

The ALI includes the NLR, so it is also an indicator of inflammation. Neutrophils activated by the immune response are complex cells capable of performing a large number of specialized functions. Experiments by Mantovani et al. have shown that overactivated neutrophils can lead to chronic inflammation and drive the expansion of innate and adaptive immune responses [19]. While neutrophils are important effectors of acute inflammation, neutrophil depletion in cardiac healing results in exacerbation of HF [20]. Lymphocytes are important immune cells involved in the pathogenesis of HF. T lymphocytes can regulate inflammation and related processes. These cells have been demonstrated to be involved in inflammation, hypertrophy, fibrosis and dysfunction of the heart [21]. Neutralization of T cells has been shown in animal models to have an attenuating effect on leukocyte recruitment and the development of cardiac hypertrophy. T-cell depletion also reduces myocardial fibrosis, thereby modifying cardiac dysfunction [22].

Our study combines ALI and GNRI to predict the prognosis of patients with HF. The risk of death is highest when the ALI is < 24.60 and GNRI < 94.41, which reminds us of the need to pay clinical attention to the nutritional assessment of the patient and to improve the prognosis of the patient by regular nutritional therapy and by aggressive anti-inflammatory treatment.

There are some limitations to this study. This study is a retrospective observational study and may have some degree of selection bias and missing data. Further prospective studies are needed to test the prognostic impact of the ALI combined with the GNRI in patients with HF. Our primary study was in patients with NYHA class III or IV. Therefore, some study results may not be available for patients with comparatively mild HF symptoms. We can collect data from patients with lower NYHA classes in the future to improve our research.

## Conclusion

Our study shows that the ALI and GNRI are independent predictors of prognosis in patients with HF. There was a mild positive linear correlation between the ALI and GNRI, and there was no interaction. The risk of all-cause mortality was highest in patients with HF when ALI < 24.60 and GNRI < 94.41. ALI and GNRI have a significant impact on the prognosis of HF patients, and the ALI combined with the GNRI can be a good predictor of the prognosis of HF patients.

## Data Availability

The datasets used and/or analysed during the current study are available from the corresponding author on reasonable request.
